# Downregulation of hsa_circRNA_0001400 Helps to Promote Cell Apoptosis Through Disruption of the circRNA_0001400–miR-326 Sponge in Cervical Cancer Cells

**DOI:** 10.3389/fgene.2021.779195

**Published:** 2021-12-17

**Authors:** Yantao Cai, Chuyu Li, Fang Peng, Shuanghong Yin, Huiyi Liang, Jiyan Su, Lin Li, Anping Yang, Hui Liu, Chuansheng Yang, Dixian Luo, Chenglai Xia

**Affiliations:** ^1^ Affiliated Foshan Maternity and Chlid Healthcare Hospital, Southern Medical University, Foshan, China; ^2^ Department of Pharmacology, Guangdong Medical University, Zhanjiang, China; ^3^ Guangdong Second Provincial General Hospital, Guangzhou, China; ^4^ School of Pharmaceutical Sciences, Southern Medical University, Guangzhou, China; ^5^ School of Medicine, Foshan University, Foshan, China; ^6^ Department of Head-Neck and Breast Surgery, Yuebei People’s Hospital of Shantou University, Shaoguan, China; ^7^ Department of Laboratory Medicine, Huazhong University of Science and Technology Union Shenzhen Hospital (Nanshan Hospital), Shenzhen, China

**Keywords:** circRNA, cervical cancer, apoptosis, phosphatidylinositol-3 kinase, Akt

## Abstract

**Background:** In recent years, circular RNAs (circRNAs) have been reported to serve as essential regulators in several human cancers. Nevertheless, the function and mechanism of circRNAs in cervical cancer remain elusive.

**Methods:** Flow cytometry assays were performed to measure cell apoptosis and cell cycle. Colony Formation and transwell chamber were performed to measure cell migration and invasion. Double luciferase reporter for gene analysis was used to detect the interaction between hsa-circRNA_0001400, miR-326, and Akt. Relative protein levels were determined by immunoblotting and relative gene levels were determined by quantitative real-time PCR. Tumor Xenograft Modeling was used to evaluate the effect of hsa_circRNA_0001400_siRNA *in vivo*.

**Results:** In the present study, we showed that hsa_circRNA_0001400 was highly expressed in cervical cancer tissues relative to in matched normal tissue. We found that hsa_circRNA_0001400_siRNA significantly promoted the apoptosis of cervical cancer cells and arrested the cell cycle and migration of cervical cancer cells. We showed that hsa_circRNA_0001400_siRNA can inhibit the protein expression of Akt and that the inhibition of miR-326 could rescue the inhibition of Akt in cervical cancer cells. We found that has-miR-326 was downregulated in cervical cancer tissues and hsa_circRNA_0001400_siRNA could increase the gene expression of has-miR-326. We also observed that hsa_circRNA_0001400_siRNA inhibited the growth and angiogenesis of SiHa xenografts in nude mice.

**Conclusion:** In conclusion, this study provides evidence that the hsa_circRNA_0001400–miR-326–Akt network promotes cervical cancer progression. Notably, our findings demonstrate the novel antitumor effects of hsa_circRNA_0001400_siRNA in cervical cancer.

## Background

Cervical cancer is the fourth leading cause of cancer death in women worldwide and is second only to lung cancer in developed regions (Small Jr et al., 2017). The 2017 American Cancer Society Guidelines noted that 12,820 women in the United States were diagnosed with invasive cervical cancer in 2016 and predicted that 4,210 of those women would die from the disease (Sawaya et al., 2019). Infection by human papillomavirus (HPV) is a major cause of cervical cancer in women. However, though HPV vaccines exist for the prevention of cervical cancer in women, they are still not widely available in developing countries and, as a result of the persistent lack of screening, a large number of cervical cancer cases in the next 50 years can be expected (Wang et al., 2020). For early cancer patients, the 5-year survival rate is 100% with surgery and, in recent years, the incidence of cervical cancer has trended toward affecting women of youngers age, making the need to retain fertility a concern. In addition, statistics show that 10–15% of patients with cervical cancer after treatment experience recurrence or metastasis, which is the main reason for the failure of cervical cancer treatment. Therefore, there is an urgent need to clarify the mechanism of cervical cancer metastasis, develop new antitumor drugs, improve the quality of life of patients with cervical cancer, and improve the 5-year survival rate (Canfell, 2019).

Circular RNAs (circRNAs) are noncoding RNAs that covalently join the 5’ and 3’ ends of linear RNA precursors via reverse splicing and play a regulatory role in many biological processes, such as cell proliferation, senescence, and apoptosis (Visci et al., 2020). CircRNAs may play an important role in tumorigenesis and development (Zhou et al., 2018). Several reports have demonstrated that the circRNA–microRNA (miRNA)–messenger RNA (mRNA) signaling pathway is involved in tumorigenesis (Zhou et al., 2018; Xu et al., 2020). In a study of tumor metastasis, circRNA had unique advantages and its ring structure was shown to determine its stability, which can greatly reduce the possibility of a mistargeting effect in clinical treatment. At the same time, many studies have also found that there are many kinds of circRNA in human body fluid, suggesting that circRNAs may be a new target for noninvasive disease diagnosis and prognosis (Zhang et al., 2018; Lu et al., 2020). Several studies to date have evaluated the roles of circRNAs and their possible mechanisms in cervical cancer andavailable research findings have indicated that circRNAs are involved in cervical tumor progression by various mechanisms, among which miRNA sponging is the most important (Kristensen et al., 2018). The study of circRNAs potentially represents a new promising strategy for diagnosis and treatment of cervical cancer.

In this study, we used RNA sequencing to analyze the differential expression of circRNA in human cervical cancer and found that hsa_circRNA_0001400 exhibited high expression in cervical cancer tissue. By silencing circRNAs with small interfering RNA (siRNA) interference, the molecular mechanism of hsa_circRNA_0001400–miR-326–Akt sponge in cervical cancer migration was elucidated from the cytological and zoological levels.

## Materials and Methods

### Cell, Reagents, and Antibodies

Human cervical cancer SiHa cells, HeLa cells, and VK2/E6E7 cells were obtained from our laboratory. Fetal bovine serum and Dulbecco’s modified Eagle’s medium culture medium were purchased from Gibco Laboratories (Gaithersburg, MD, United States). Additionally, Transwell Cell (8.0 μM; Corning, Corning, NY, United States); Crystal Violet (Beijing Regan Biotechnology Co., Ltd. Beijing, China); and primary antibodies including glyceraldehyde 3-phosphate dehydrogenase (GAPDH) (cat.#5174), Akt (cat.#4691), Phospho-Akt (Ser473) (cat. #4060), Phospho-PI3 Kinase p85 (Tyr458) (cat.#17366)and PI3K (cat.#4249) as well as the second antibody (Anti-rabbit IgG, cat.#7074) were purchased from Cell Signaling Technology (Danvers, MA, United States). Lipofectamine 2000 was purchased from Invitrogen Corporation (Carlsbad, CA, United States). We designed hsa_circRNA_0001400 primer and siRNA according to the has_circRNA_0001400 sequence listed in the CircRNA database using the methods described in a previous study (Dalmer and Clugston, 2019). The primers of hsa_circRNA_0001400, miR-326, and hsa_circRNA_0001400_siRNA were synthesized by Genecfps Corporation (Shanghai, China). Finally, the microRNA kit used to analyze microRNAs was purchased from FulenGene Biology Co., Ltd. (Shanghai, China). The sequences of the primers were as follows:

hsa_circRNA_0001400-F:ATGTCTGTTAGTGGGGCTGA;
hsa_circRNA_0001400-R:TATCTGCTACCATCGCCTTT;
miR-326 mimic: CCUCUGGGCCCUUCCAG;
miR-326 inhibitor: CTG​GAG​GAA​GGG​CCC​AGA​GG
has-miR-326-F: CCT​CTG​GGC​CCT​TCC​TCA​G;
has-Akt2-F:CTCAGCATCAACTGGCAGGA;
hsa-Akt2-R: GTG​ATG​GAC​TGG​GCG​GTA​AA;
hsa_circRNA_0001400_siRNA-F:5′-AGUAGCAGCGAAUGCUGAUGUUU-3’;
hsa_circRNA_0001400_siRNA-R: 5′-ACA​UCA​GCA​UUC​GCU​GCU​ACU​UU-3’

### CircRNA Sequencing

In this study, six cervical cancer tissue samples were collected from January 2019 to December 2019 at Foshan Maternity and Child Healthcare Hospital. All samples were collected after receiving signed informed consent forms from patients and their families and the approval of the Ethics Committee of Foshan Maternity and Child Healthcare Hospital (No. FSFY-MEC-2018-061). Specimens from patients were diagnosed by pathological examination with carcinoma *in situ* of the cervix. The patient did not receive any radiation, chemotherapy, or other anticancer treatment prior to the operation. The tumor tissue was removed and transferred to the laboratory in an icebox at a low temperature. After cleaning three times with phosphate-buffered saline (PBS), the tumor tissue was stored in liquid nitrogen for further extraction of total RNA. Additionally, specimens collected from 2 cm away from the edge of tumor tissue were confirmed to be normal tissue by pathological examination. There was no atypical hyperplasia or infiltration of cancer tissue.

The sample library was built an ILUMIMA kit (Illumina, Inc. San Diego, CA, United States) and the original data were sequenced using HiSeq 4000 (Illumina, Inc. San Diego, CA, United States). The simple procedure used was as follows: the sample RNA was reverse-transcribed into complementary DNA; then, poly-A was added at the 3’ end, then the sample was connected to the sequencing connector of the kit, and polymerase chain reaction (PCR) amplification was performed. Finally, HiSeq 4000 was used to detect the fault. The original image files of Illumina high-throughput sequencing were base-called and transformed into original sequence sequences (sequenced reads) for subsequent analysis—that is, a collection of original sequences, called raw data. The analysis results are presented in FASTQ files (FQs for short). Based on the long noncoding RNA length threshold, potential assembled transcripts shorter than 200 bp were filtered out. For the assembled transcripts with only one exon, the transcripts with FPKM values of at least 2, and those with multiple exons, the transcripts with FPKM values of at least 0.5 were kept. When more than three samples were present, at least two samples were required to meet the screening criteria. The unknown transcripts were assembled by the Stringtie software for further coding ability filtering and the CPC, CNCI, and HMMER software programs were used for coding ability prediction, while FASTQC (version 0.11.2), fastp (version 0.14.0), Top Hat (version 2.0.13), Lemma (version 3.32.10) and Edger (version 3.18.1) were used for quality-control analysis.

### Kyoto Encyclopedia of Genes and Genomes (KEGG) Analysis

Cluster analysis based on the functional enrichment of varying groups of differentially expressed circRNA was performed to investigate the potential associations and differences in specific functions (KEGG pathway) (Xia et al., 2020a). We first collected functional classification information enriched by circRNA grouping and the corresponding enriched *p*-values, then filtered out functional classifications that were significantly enriched (*p* < 0.05) in at least one circRNA grouping. First, the *p*-value data matrix was transformed using the log10 logarithm; then, the transformed data matrix was transformed by Z transformation for each function classification. Finally, hierarchical clustering (Euclidean distance, average join clustering) was used to analyze the data set after Z transformation. Clustering relationships were visualized using heat maps drawn by the function heatmap 2 in the R language package gplots (R Foundation for Statistical Computing, Vienna, Austria).

### Cell Cycle

According to the methods described in a previous study (Xia et al., 2020b), when the cell density of HeLa and Siha cells was up to 70%, fresh medium was used. In preparing for transfection, 10 μL of siRNA (10 μL of negative control or 5 μL of siRNA + 5 μL of inhibitor) was diluted in 250 μL of Opti-MEM, while 10 μL of Lipofectamine 2000 transfection reagent was diluted in another 250 μL of Opti-MEM, with the transfection reagent left to rest for 5 min. The mixed solution was added into the holes for transfection and the complete medium was changed after 12 h. After being cultured for 48 h, the cells were removed from the incubator, the medium was discarded, the cells were rinsed with PBS, 0.25% trypsin was added, and the cells were digested at 37°C for 1–2 min. Then, 1–2 ml of medium was added to complete digestion and the cells were blown down with a pipette gun, transferred to a 15-ml centrifuge tube at 1,000 rpm, and centrifuged for 5 min and the supernatant was discarded. The cells were then resuspended and transferred to a sterile Eppendorf enzyme-free tube at 1.5 ml, then centrifuged for 2 min at 4°C and 500 g, and the supernatant was discarded. Precooled PBS was added again, under the same conditions of centrifugation, and the supernatant was discarded. We added 250 μL of precooled PBS, then 750 μL of precooled anhydrous ethanol, which were gently blown on and mixed at 4°C overnight, then fixed. Cells were centrifuged at 4°C and 500 g for 2 min, and then, the supernatant was discarded and washed with precooled PBS. Cells were centrifuged again at 4°C and 500 g for 2 min; then, the supernatant was discarded and cells were washed again. After washing, the cells were resuspended by PBS with 300–500 μL of supernatant; the final concentration of propidium iodide (PI) and RNaseA was 50 g/ml. Finally, the cells were warmed at 37°C for 30 min and cell-cycle distribution was measured and analyzed by flow cytometry. The flowcytometry in our laboratory is FACS Canto^TM^ II (Becton, Dickinson and Company, United States). The software we applied to process apoptotic data is called DIVA (Version 8.0).

### Cell Apoptosis

As described in the methods of a previous study (Xia et al., 2018), HeLa and Siha cells (1 × 10^6^) were planted in six-well plates. When the cell density was 70%, the fresh medium was changed. In preparing for transfection, 10 μL of siRNA (10 μL of negative control or 5 μL of siRNA + 5 μL of inhibitor) was diluted in 250 μL of Opti-MEM, while 10 μL of Lipofectamine 2000 transfection reagent was diluted in another 250 μL of Opti-MEM, with the transfection reagent left to rest for 5 min. The mixed solution was added into the holes for transfection and the complete medium was changed after 12 h. The cells were incubated for 48 h, then digested with trypsin free of EDTA, gently beaten and transferred to a 1.5-ml Eppendorf tube, and centrifuged for 5 min at 1,000 rpm. We used Dead Cell Apoptosis Kit with Annexin V FITC and PI (ThermoFish, V13242) to measure cell apoptosis. FITC-Annexin V was Soluted in 25 mM HEPES, 140 mM NaCl, 1 mM EDTA, pH 7.4, 0.1% bovine serum albumin (BSA). The supernatant was discarded and cells were rinsed gently with an appropriate amount of PBS for 5 min at 1,000 rpm three times, then gently stirred with 500 μL of binding buffer and mixed with 5 μL of annexin V-FITC and incubated at room temperature under light for 15 min. Finally, they were mixed with 1 μL of the 100 μg/ml PI under light for 5 min and apoptosis was detected by flow cytometry.

### Cell Migration

According to the methods described in a previous study (Abere et al., 2020), when the cell density of HeLa and Siha cells was 70%, the fresh medium was changed. In preparing for transfection, 10 μL of siRNA (10 μL of negative control or 5 μL of siRNA + 5 μL of inhibitor) was diluted in 250 μL of Opti-MEM, while 10 μL of Lipofectamine 2000 transfection reagent was diluted in another 250 μL of Opti-MEM, with the transfection reagent left to rest for 5 min. The mixed solution was added into the holes for transfection and the complete medium was changed after 12 h. After 24 h, the supernatant was discarded, cells were rinsed again with 2 ml of PBS and digested with 1 ml of trypsin for 2 min. Next, 1 ml of complete medium was added to stop digestion, cells were transferred to an Eppendorf tube and centrifuged at 500 g at room temperature for 2 min, and the supernatant was discarded. Then, we added 2 ml of basic medium, blew with spearhead, prepared the cell suspension, and counted the cells. Next, 500 μL of basal medium was added into a Transwell pore plate (Corning Inc. Corning, NY, United States) and the membrane was soaked for 30 min, then activated. After activation, the basal medium was discarded, and 400 μL of complete medium was added in the lower chamber, gently placed into the upper chamber, and 1 × 10^5^ cells were added. We cultured the cells for 36 h, discarded the plate of upper-chamber culture medium, and rinsed it gently twice with PBS. The medium in the lower chamber was also discarded, 600 μL of formaldehyde was added, 200 μL was added in the upper chamber and fixed at room temperature for 15 min. After fixation, the formaldehyde was discarded, washed gently three times with PBS, and then dyed with 1% crystal violet.

### Colony Formation

Cell suspension was prepared according to a previous study (Abere et al., 2020), where the concentration of cells was adjusted by the cell culture medium. A total of 1,000 cells were placed into the cell culture medium and inoculated with 5 ml of cell culture medium; then, hsa_circ_0001400_siRNA and its control siRNA were added and incubated at 37°C and 5% CO_2_ for10 days, withthe medium replaced with fresh medium followingany pH changes. When visible clones appeared in the dish, we discarded the culture medium and used PBS solution to carefully soak and wash the cells twice. The colony was fixed with methanol for 20 min. Then the methanol was discarded and the colony was allowed to air-dry. Only then were the counts performed. Then, Giemsa solution was used to dye the colony for 30 min beforebeing washed off slowly with running water. The percentage of colony formation was calculated according to the following equation: % colony = (the number of colonies/total cells number) × 100%.

### RNA Extraction and Quantitative Real-Time PCR

We discarded the cell culture solution, washed with PBS twice, added 1 ml of TRIzol reagent, blew gently until the cells fell off, inhale a 1.5-ml Eppendorf tube, and rested for 5 min. Then, we added 200 μL of chloroform, shook vigorously by hand for 15 s, left at room temperature for 23 min, and centrifuged at 4°C and 12,000 g for 15 min. We carefully transferred the upper aqueous phase to a new Eppendorf tube, without excessive volume (about 400–500 μL). We added isopropanol of equal volume into the upper aqueous phase mixture, gently mixedisopropanol and the upper aqueous phase mixture five times, and left the mixture at room temperature for 10 min. After 12,000 g of centrifugation at 4°C for 10 min, and 1 ml of 75% ethanol (0.75 ml of anhydrous ethanol + 0.25 ml of diethylpyrocarbonate water, now mixed) were added, then cells were washed twice, swirled, and mixed completely. Finally, we removed the supernatant, air-dried the RNA for 10 min, added 20 μL of diethylpyrocarbonate water, blew several times, and measured the concentration.

The all-in-one miRNA qRT-PCR kit (no. AOMD-Q020; GeneCopoeia, Guangzhou, China) was used. The reaction conditions were set according to the standard procedure given by the kit. The specific procedures were as follows: the components were mixed according to the configuration table, followed by instant centrifugation, incubation at 37°C for 60 min, incubation at 85°C for 5 min, reverse transcription, predenaturation at 95°C for 1 minute, denaturation at 95°C for 10 s, annealing at 53°C for 10 s, and acceptance at 72°C for 10 s, then repeat for 40 cycles, with analysis using the Roche LC96 default melting curve (Roche Holding, Basel, Switzerland). The amplification specificity of the miRNA in each sample was analyzed by the melting curve. The comparative cycle threshold (2^−ΔΔT^) was used to evaluate the miRNA expression of the target. For mRNA detection, qRT-PCR was reverse-transcribed using the Bestar qPCR RT Kit (No. 2220, DBI Bioscience) and Bestar SYBR Green qPCR Mix Master (No. 2043, DBI Bioscience) for gene detection. The reaction conditions were set according to the standard procedure given by the kit as follows: the components were mixed according to the configuration table, followed by instant centrifugation, incubation at 37°C for 15 min, incubation at 98°C for 5 min, reverse transcription, predenaturation at 95°C for 2 min, denaturation at 95°C for 10 s, annealing at 53°C for 30 s, and acceptance at 72°C for 30 s, then repeat for 40 cycles, with analysis using the Roche LC96 default melting curve. The specificity of the mRNA amplification in each sample was analyzed by the melting curve. The comparative period threshold (2−ΔΔCt) was used to assess target mRNA expression.

### Double Luciferase Reporter for Gene Analysis

According to the methods described in a previous study (Abere et al., 2020), 293T cells (37°C, 5% CO_2_) were inoculated into a 24-well plate with 1.5 × 10^5^ cells per well for a total volume of each well of 500 ml, then cultured in a 37°C incubator for 24 h. Dilution of 1.5 μL of miR-Mimic (inhibitor, negative control) with 25 ml of Opti-MEM medium diluted with 0.6 μg of the target gene 3’UTR double-report gene vector (50 ml) was performed. Also, Opti-MEM medium was diluted with 2 ml of Lipofectamine 2000 reagent and the mixture was mixed for 5 min, shaken gently, and left to rest for 20 min. Before the transfection mixture was added into cells, 100 μL of medium was sucked out per hole; then, the 100 ml mixture was added and, finally, the total volume of each hole was 500 ml, with three double holes in each group. The fresh medium of 500 μL was changed after 8 h. After the lysate of reporter cells had been thoroughly thawed and mixed, 200 μL of lysate of reporter cells was added to lyse the cells. Next, centrifugation at 4°C and 10,000 g for 1 minute was performed, keeping the supernatant. For each sample, 100 μL of serum was taken from the top of the sample and 100 μL of Firefly luciferin was added to the mixture to determine the relative light unit. Reporter gene cell lysate was used as a blank control. After completion of the above Firefly luciferin procedure, the relative light unit value was determined by adding 100 μL of harennin luciferase detection working fluid and mixing it in. The relative fluorescence expression of each group was calculated.

### Western Blotting

According to the methods described in a previous study (Abere et al., 2020), the samples were taken from −80°C, and 4× volume cracking buffer solution (8 m urea, 1% protease inhibitor) was added to the samples. RIPA containing protease inhibitors was used to extract protein and cells were lysed for 30 min. Centrifugation was performed at 4°C and 12,000 g for 10 min, the cell fragments were removed, the supernatant was transferred to a new centrifuge tube, and the protein concentration was determined by bicinchoninic acid (BCA) protein assay kit (Novagen, Madison, WI, United States). The prepared buffer was poured into the electrophoresis tank and the precooled protein sample and protein maker were slowly added into the gel pore. We ran the gel at 80 volts (V), then switched to 110 V until the bromophenol blue dye moved to the bottom of the gel and stopped electrophoresis. The sandwich splint was placed in the electrophoresis tank at 4°C and 250 mA of constant current was applied for 60 min; then, 5% skimmed milk powder was sealed at room temperature for 2 h, the first reactant was added and incubated overnight at 4°C, and the second reactant was incubated at room temperature for 1.5 h. The PVDG membrane was incubated with electro-chemiluminescence for 3 min at room temperature and imaged using the Bio-Rad gel system. We use ImageJ to analyze immunoblot lane intensity. Calculation results from each independent experiment were presented by folds to control group.

### Tumor Xenograft Modeling and *In Vivo* Experiments

BALB/C nude mice (3–4 weeks old) were purchased from the Laboratory Animal Center of Guangdong Province. Weighing about 15–17 g, the mice were injected with 0.1 ml of Siha single-cell suspension (containing about 5×10^6^ cells) in serum-free medium to disinfect the skin near the armpit of the left forelimb. When a rice-size nodule appeared (1–2 mm^3^) in nude mice (about 1 week after injection), the xenograft model of cervical cancer was believed to be successfully established. The subcutaneous tumors of the left forelimb of nude mice were measured with electronic Vernier calipers. When the diameter of the tumor was about 0.3–0.5 cm, the nude mice were numbered using a random number table method and randomly divided into five groups (model group, negative control group, high-dose group, medium-dose group, and low-dose group), with six mice in each group. The growth of nude mice and their tumors were closely observed every day, focusing on whether the tumors were inflamed or ulcerated, whether the skin of nude mice had luster, and whether their mental state was good were observed. The maximum and minimum diameters of subcutaneous tumors in nude mice were measured with the Vernier scale every 2 days and the tumor volume was calculated according to the following: V = 0.5 × A × B^2^ (where V = tumor volume, A = tumor maximum diameter, and B = tumor minimum diameter). In this experiment, the hsa_circRNA_0001400_siRNA was given once a day for 4 days. After drug treatment, the nude mice were killed by cervical dislocation and the subcutaneous tumor was quickly peeled off on ice. The weight of each tumor was weighed by electronic balance and some tumor tissue was cut and placed into a cryopreservation tube and stored in liquid nitrogen for subsequent experimental analysis.

### Statistical Analysis

Using the Statistical Package for the Social Sciences version 16.0 statistical software (IBM Corporation, Armonk, NY, United States) for data analysis, data were compared between two groups using an independent *t*-test, while comparisons between more groups were made using multifactor analysis of variance. *p* < 0.05 indicated a significant difference, while *p* < 0.01 suggested a very significant difference.

## Results

### The Differential Expression of circRNA in Cervical Cancer

**FIGURE 1:**
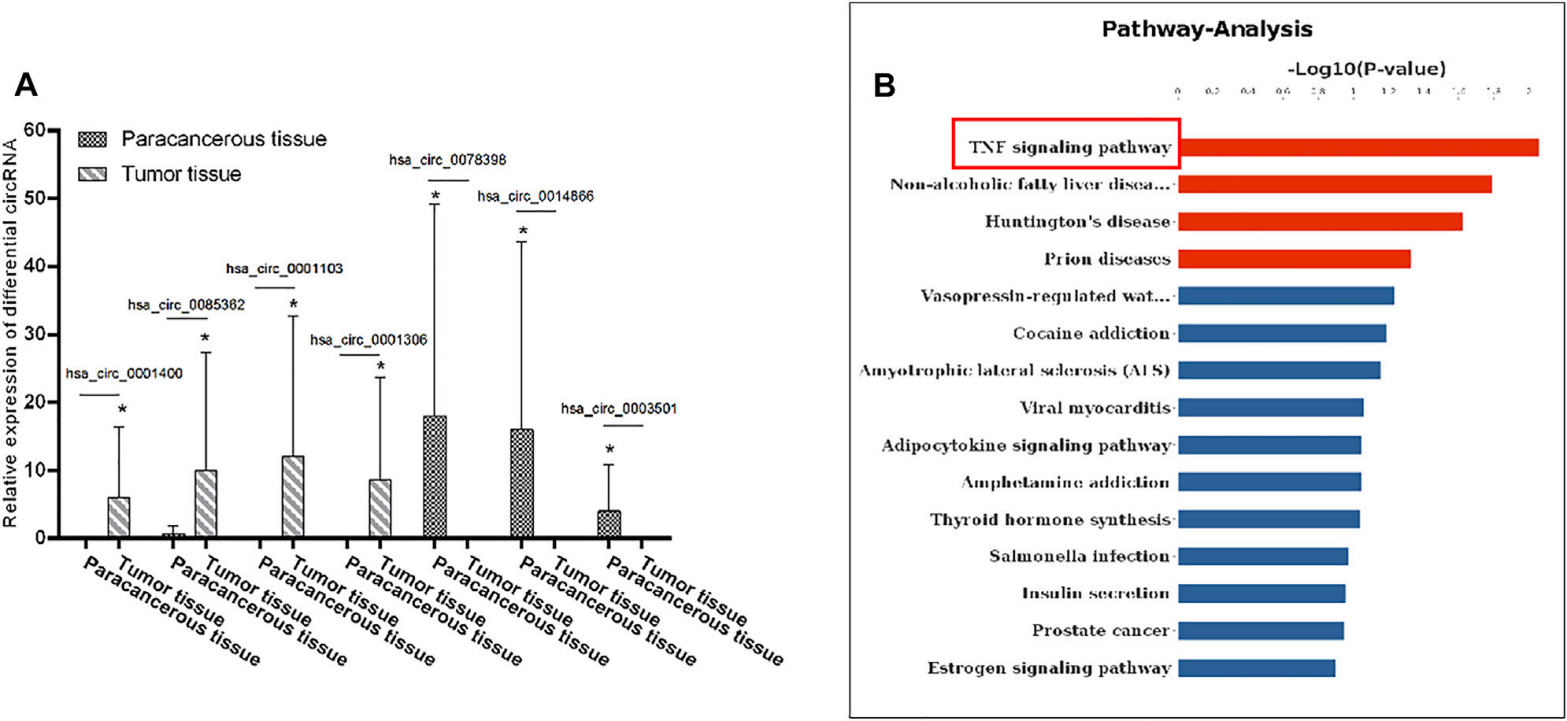
Differential expression levels of circRNA in cervical cancer: **(A)** The expression levels of hsa_circRNA_0001400, hsa_circRNA_0085362, hsa_circRNA_0001103, and hsa_circRNA_0001306 were significantly increased and those of hsa_circRNA_0078398, hsa_circRNA_0014866, and hsa_circRNA_0003501 were significantly decreased. B) KEGG was used to analyze the signal pathway involved in the differential expression of circRNA. Data are expressed as mean ± standard deviation, *n* = 3, *p < 0.05 and **p < 0.01 vs. control group.

In order to explore the role of circRNA in the pathogenesis of cervical cancer, we used an RNA sequencing method to search for differential circRNA. Specifically, we used the differential expression multiple of more than 1.2-fold as the standard for screening and found that, as compared with the adjacent tissues, a total of 48 circular RNAs were expressed, of which 28 were upregulated and 20 were downregulated. Notably, hsa_circRNA_0001400, hsa_circ_0085362, hsa_circ_0001103, and hsa_circ_0001306 were significantly increased, while hsa_circ_0078398, hsa_circ_0014866, hsa_circ_0003501 were significantly decreased (Figure 1A).

We first used bioinformatics to analyze the results of RNA sequencing. GO is an important bioinformatics analysis method and tool used to express the attributes of genes and gene products. GO notes can be divided into three categories: biological process, cellular components, and molecular function, which explain the biological function of proteins from different perspectives. We analyzed the distribution of differentially expressed circRNA in GO secondary annotation. According to the classification results of biological processing, we found that these circRNAs are mainly involved in the apoptosis signal pathway through death receptors (Supplementary Figure S1A); according to the classification results of cellular components, these circRNAs mainly belong to the interleukin complex and protein kinase complex (Supplementary Figure S1B), while, according to the classification results from molecular function, these circRNAs mainly have binding functions (Supplementary Figure S1C). KEGG is an information network connecting known molecular interactions, such as metabolic pathways, complexes, and biochemical reactions. The KEGG pathway mainly includes metabolism, genetic information processing, environmental information processing, cell processes, human diseases, drug development, and so on. Protein domain refers to some components that appear repeatedly in different protein molecules. The similar sequence, structure, and function of proteins is the unit in evolution. The length of the domain is usually between 25 and 500 amino acids. From the enrichment of the KEGG pathway, we found that the differentially expressed circRNA mainly regulates the tumor necrosis factor signaling pathway (Figure 1B). These results suggest that circRNA can regulate the apoptosis signaling pathway and combine with downstream signaling molecules to participate in the pathogenesis of cervical cancer.

Hsa-circRNA_0001400_siRNA promotes cervical cancer cell apoptosis and arrest of thecell cyclein the G2 phase.

**FIGURE 2:**
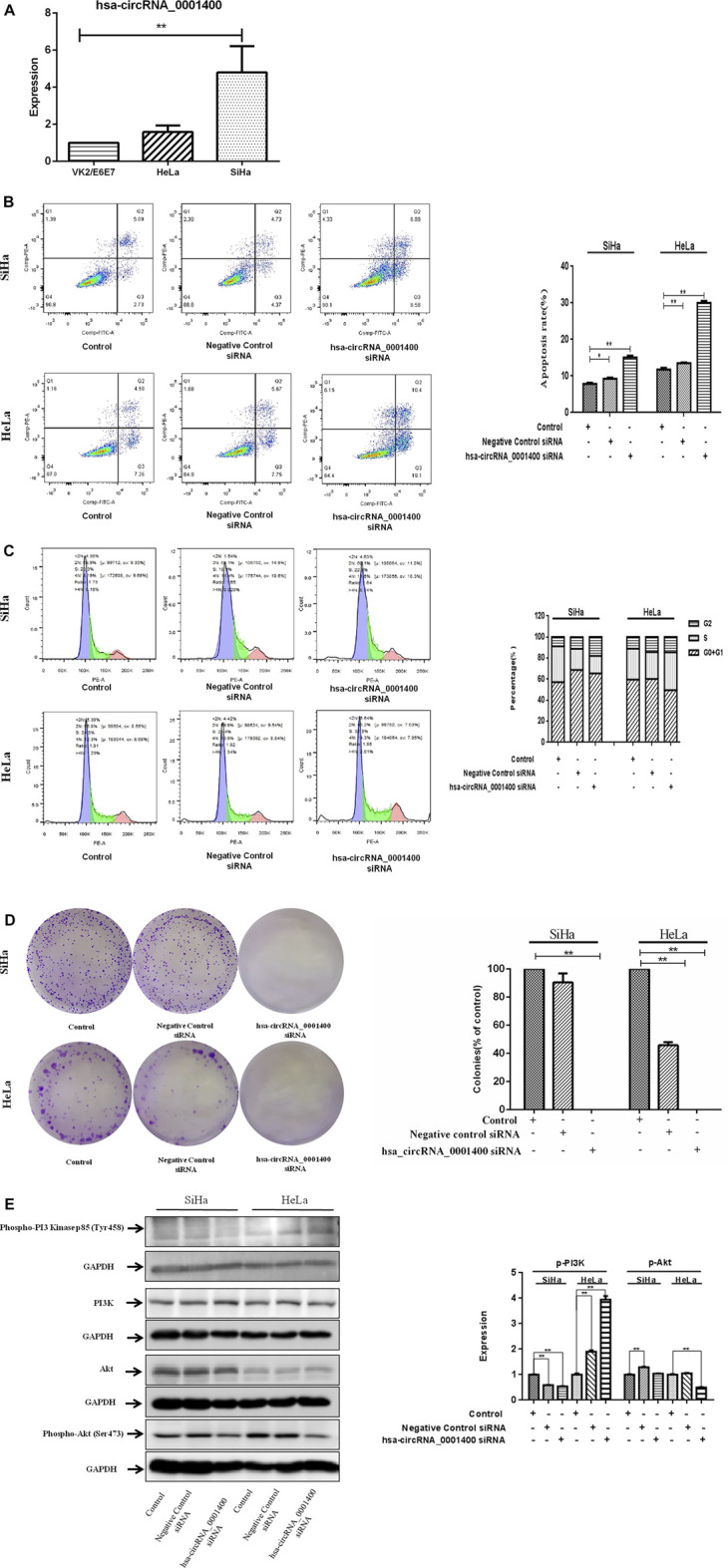
Hsa-circRNA_0001400_siRNA promotes the apoptosis of cervical cancer cells and causes G1-phase arrest of the cell cycle. **(A)** hsa_circRNA_0001400 in human vaginal epithelial, cervical cancer SiHa, and HeLa cell lines. **(B)** The effect of hsa_circRNA_0001400_siRNA on the apoptosis of cervical cancer cells. **(C)** The effect of hsa_circRNA_0001400_siRNA on the cell cycle of cervical cancer cells. **(D)** The effect of hsa_circRNA_0001400_siRNA on cervical cancer colony formation. **(E)** The effects of hsa_circRNA_0001400_siRNA on Akt, Phospho-Akt (Ser473), PI3K, and Phospho-PI3 Kinase p85 (Tyr458) protein expression in cervical cancer cells. Data are expressed as mean ± standard deviation, *n* = 3, *p < 0.05 and * *p < 0.01 vs. control group.

Our sequencing results showed that hsa_circRNA_0001400 is a highly expressed circRNA in tumor tissues. Previous studies showed that hsa_circ_0001400 was up-regulated upon Kaposi’s sarcoma herpesvirus (KSHV) infection in the microarray expression profiling (Takanobu Tagawa, et al., 2018). Thus, we selected hsa_circRNA_0001400 for follow-up study. We confirmed hsa_circRNA_0001400 to be highly expressed in cervical cancer cell lines (Figure 2A). Unlimited proliferation and permanent cell division are the characteristics of tumor cells. Therefore, it is an important strategy to inhibit tumor growth by inhibiting tumor cell proliferation, promoting tumor cell apoptosis, and blocking tumor cell division. We used hsa_circRNA_0001400_siRNA to trigger interference expression of hsa_circRNA_0001400 by flow cytometry. We found that hsa_circRNA_0001400_siRNA can significantly promote the apoptosis of SiHa and HeLa cells and arrest the division of both cell types in the G2 phase (Figures 2B,C). We also analyzed the colony formation of cervical cancer cells under the presence of hsa_circRNA_0001400_siRNA. We found that hsa_circRNA_0001400_siRNA can significantly suppressthe colony formation of SiHa and HeLa cells (Figure 2D). The PI3K-Akt signaling pathway is an important signal pathway of tumor proliferation. Akt is at the center of the signal pathway network, responsible for a variety of signal transduction functions, and is a key molecule in the PI3K–Akt signaling pathway. We analyzed the effect of hsa_circRNA_0001400_siRNA on PI3K, Phospho-PI3 Kinase p85 (Tyr458), Akt and Phospho-Akt (Ser473) in cervical cancer cells. Our results showed that hsa_circRNA_0001400_siRNA can inhibit the expression of p-Akt in SiHa and HeLa cells. The expression of p-PI3K was not affected by hsa_circRNA_0001400_siRNA. Interestingly, these results showed that the target gene of microRNA adsorbed by hsa_circRNA_0001400 might be Akt and not to be PI3K (Figure 2E).

### The Direct Interaction Between hsa-circRNA_0001400, miR-326, and Akt

**FIGURE 3:**
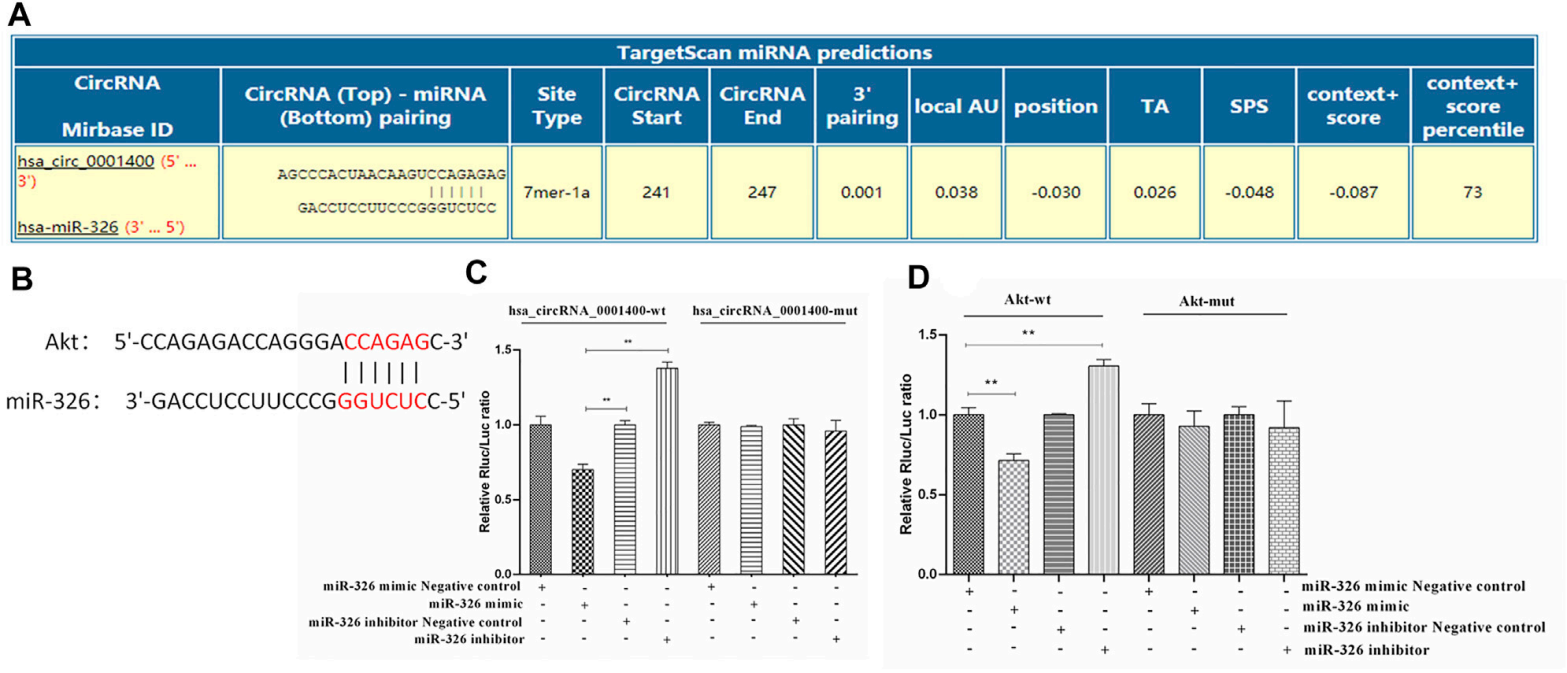
Direct interaction of hsa-circRNA_0001400, miR-326, and Akt. **(A)** Bioinformatics prediction of the direct interaction sites of hsa_circRNA_0001400 and miR-326. **(B)** Bioinformatics prediction of the direct interaction of miR-326 and Akt. **(C)** The direct interaction between hsa_circRNA_0001400 and miR-326 was analyzed by double luciferase reporter gene analysis. **(D)** The direct interaction between miR-326 and Akt was analyzed by double luciferase reporter gene analysis. Data are expressed as mean ± standard deviation, *n* = 3, *p < 0.05 and * *p < 0.01 vs. control group.

Because of its spongy function, circRNA can adsorb microRNA and regulate the function of microRNA target genes (Liu et al., 2019; Zhou et al., 2019; Yang et al., 2021). We first predicted the binding sites of miR-326, Akt, and hsa_circRNA_0001400 by bioinformatics. Our prediction results showed that the binding sites of hsa_circRNA_0001400 and miR-326 were located at 241 to 247 of the 5′ end of the circular RNA, while the binding target of mir-326 with AKT2 was located at positions 2,224 to 2,234 in the AKT2 mRNA 3′UTR region (www.https://circinteractome.nia.nih.gov/) (Figures 3A,B). We then verified the direct interaction between hsa_circRNA_0001400 and miR-326, miR-326 and Akt by luciferase reporter gene analysis. In addition, we mutated the region from position 241 to 247 at the 5′ end of the circular RNA and position 2,224 to 2,234 at the AKT2 3′UTR region in 293T cells. Our results showed that direct interaction between hsa_circRNA_0001400 and miR-326, as well as miR-326 and Akt, has disappeared (Figures 3C,D).

### miR-326 Rescues the Action of hsa-circRNA_0001400_siRNA

**FIGURE 4:**
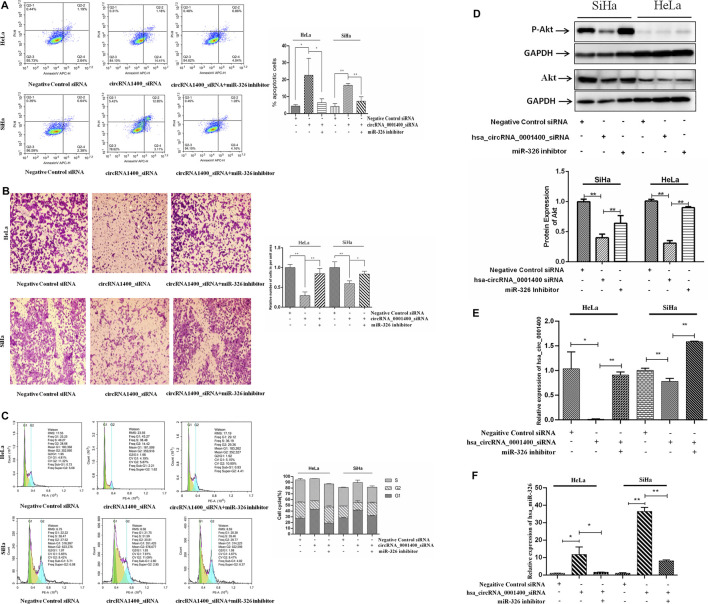
Hsa_circRNA_0001400 targeting miR-326 rescue experiment. **(A)** Apoptosis recovery test; **(B)** cell migration recovery test; **(C)** cell cycle recovery test; and **(D)** Akt and Phospho-Akt (Ser473) expression. **(E)** The effect of miR-326 inhibitor rescue of hsa_circRNA_0001400_siRNA on hsa_circRNA_0001400. **(F)** The effect of miR-326 inhibitor rescue of hsa_circRNA_0001400_siRNA on miR-326. Data are expressed as mean ± standard deviation, *n* = 3, *p < 0.05 and * *p < 0.01 vs. control group.

CircRNAs have a variety of biological characteristics (Hong et al., 2019). The mechanism of action of circRNAs is mainly focused on the sponge adsorption of miRNA. The results show that there are miRNA binding sites on circRNAs, which act as miRNA sponges by adsorbing miRNAs (Stanley et al., 2014; An et al., 2017). CircRNA contains a large number of miRNA response elements, which can bind miRNAs competitively to prevent complementary pairing with the 3′UTR region of downstream target mRNA and regulate mRNA gene expression (Tsikouras et al., 2016). We next verified that hsa_circRNA_0001400 was able to adsorb miR-326 on the sponge. When hsa_circRNA_0001400 was silenced by hsa_circRNA_0001400_siRNA, the sponge of hsa_circRNA_0001400–miR-326 was destroyed and miR-326 was transformed from hsa_circRNA_0001400–miR-326 sponge and was released. Also, the target of miR-326 was inhibited, the apoptosis of tumor cells increased (Figure 4A), migration was inhibited (Figure 4B), the number of G1-phase cells increased, and the cell division rate was slowed down (Figure 4C). When we used miR-326 inhibitor at the same time, the antitumor effect of free miR-326 released from the sponge was inhibited and tumor cell apoptosis decreased again (Figure 4A), cell migration increased (Figure 4B), the number of G1-phase cells decreased, and cells entered a rapid division and proliferation phase (Figure 4C). We also detected the protein expression of Akt and found that Akt expression decreased when hsa_circRNA_0001400 was silenced alone by hsa_circRNA_0001400_siRNA. Once miR-326 inhibition was combined with hsa_circRNA_0001400_siRNA, the Akt expression increased, which was consistent with the conclusion of flow cytometry (Figure 4D). Furthermore, we measured the expression of hsa_circRNA_0001400 under the condition of hsa_circRNA_0001400_siRNA or miR-326 inhibition. Our results showed that the expression of hsa_circRNA_0001400 decreased when hsa_circRNA_0001400_siRNA was used alone. The expression of hsa_circRNA_0001400 increased when hsa_circRNA_0001400_siRNA was combined with miR-326 inhibition (Figure 4E). We also tested the expression of miR-326 under the condition of hsa_circRNA_0001400_siRNA or miR-326 inhibition. Our results showed that the expression of miR-326 increased when hsa_circRNA_0001400_siRNA was used alone. The expression of miR-326 decreased when hsa_circRNA_0001400_siRNA was combined with miR-326 inhibition (Figure 4F). These results suggest that hsa_circRNA_0001400_siRNA could destroy the sponge of hsa_circRNA_0001400–miR-326. When the hsa_circRNA_0001400–miR-326 sponge was damaged, miR-326 was released from the sponge and suppressed the expression of Akt following the inhibition of p-Akt.

Hsa-circRNA_0001400_siRNA suppresses the growth of cervical cancer cell xenograft, decoying the hsa-circRNA_0001400–miR-326 sponge.

**FIGURE 5:**
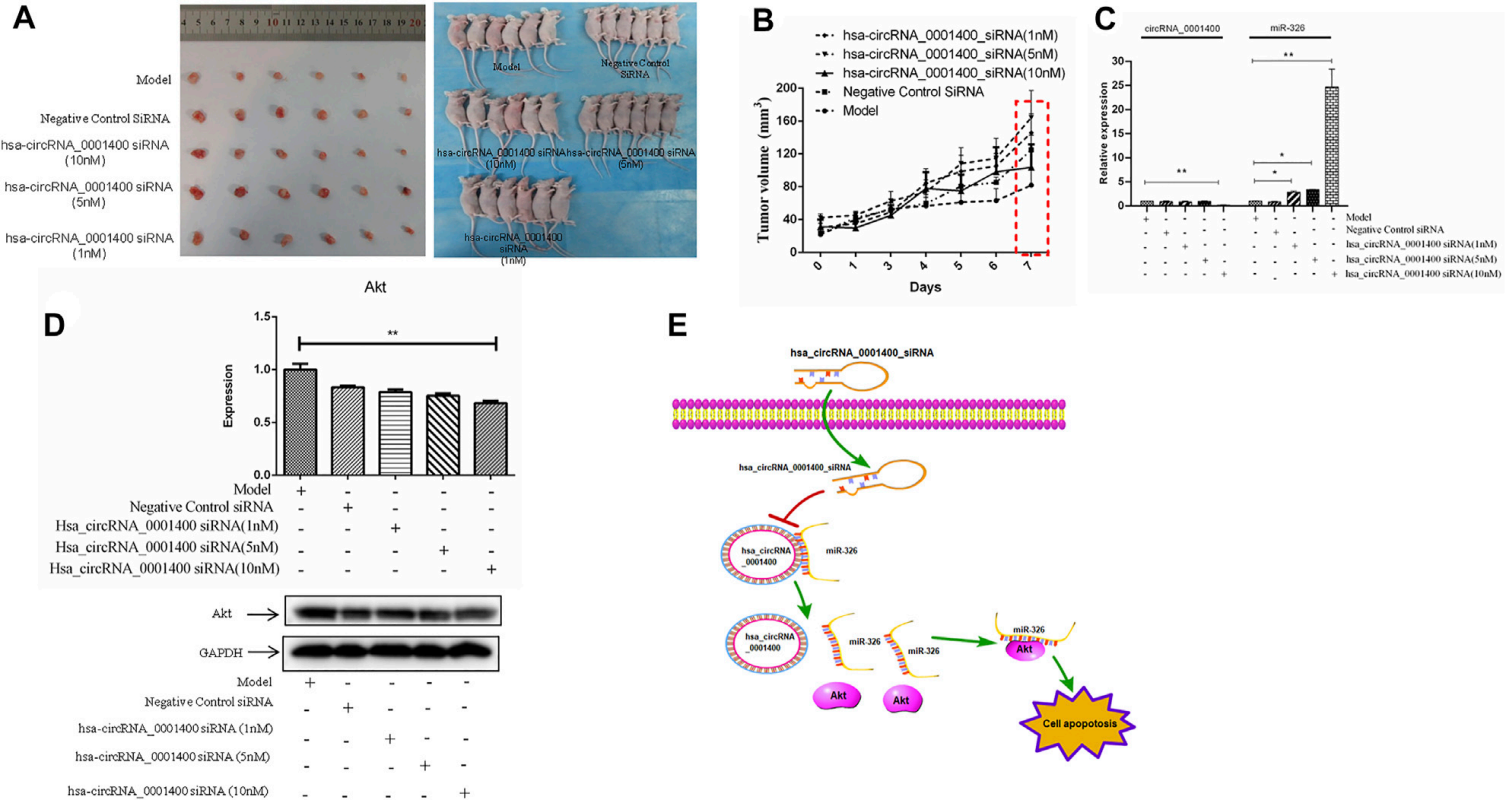
Hsa-circRNA_0001400_siRNA inhibits the growth of cervical xenograft tumors. **(A)** Tumor volume; **(B)** tumor growth curve; **(C)** the expression levels of hsa-circRNA_0001400 and miR-326; **(D)** the expression of Akt in tumor tissue; and **(E)** the pattern of action. Data are expressed as mean ± standard deviation, *n* = 3, *p < 0.05 and * *p < 0.01 vs. control group.

The animal model of xenotransplantation of tumor cells has been widely used in the screening and evaluation of antitumor drugs because of its easy construction, high tumor formation rate, and short experimental cycle (Xia et al., 2020a). To study the effect of hsa_circRNA_0001400_siRNA on tumors *in vivo*, we established a BALB/C nude mouse model of heterotopic tumor using SiHa cells. After successful modeling, 1, 5, and 10 nm of hsa_circRNA_0001400_siRNA were used in tumor mice. As compared with the model group, 10 nm of hsa_circRNA_0001400_siRNA was injected into the tumor-bearing mice for 7 days. The growth of tumors in the siRNA group was slower than that in the control group (Figures 5A,B). The expression of hsa_circRNA_0001400 and Akt decreased, while the expression of miR-326 increased (Figures 5C,D). These results suggest that hsa_circRNA_0001400_siRNA destroyed the hsa_circRNA_0001400–miR-326 sponge and may have inhibited the growth of cervical cancer xenograft in BALB/C nude mice (Figure 5E).

## Discussion

We found the expression levels of hsa_circRNA_0001400, hsa_circRNA_0085362, hsa_circRNA_0001103, and hsa_circRNA_0001306 were significantly increased in tumor tissues by RNA sequencing. We chose hsa_circRNA_0001400 to study further and found that hsa_circRNA_0001400_siRNA can promote the apoptosis of SiHa and HeLa cells and arrest the division of SiHa and HeLa cells in the G2 phase. Hsa_circRNA_0001400–miR-326–Akt sponge plays an important role in cervical cancer metastasis. Hsa_circRNA_0001400_siRNA destroys hsa_circRNA_0001400–miR-326 sponge, inhibiting Akt, promoting tumor cell apoptosis, and suppressing cervical cancer metastasis.

Although the main cause of cervical cancer is HPV infection, the availability of HPV vaccines can bring good news to patients with cervical cancer. At present, however, the HPV vaccine is mainly used to prevent tumors and a therapeutic vaccine has not yet come onto the market (Hu and Ma, 2018). At present, surgery and chemotherapy are still the main treatments. However, 10–15% of patients with cervical cancer relapse or experience metastasis after treatment, which are the primary reasons for the failure of cervical cancer treatment (Moldovan et al., 2019; Xu et al., 2019). With the development of high-throughput sequencing technology, a large number of circRNAs have been discovered and confirmed to have functions of posttranscriptional regulation (Arnaiz et al., 2019; Yu C. et al., 2019). CircRNAs have gradually attracted attention. Studies have confirmed that circRNAs affect the occurrence, development, invasion, and metastasis of tumors mainly through escaping growth inhibition and apoptosis; activating invasion, metastasis, and angiogenesis processes; and maintaining proliferation signals (Verduci et al., 2019; Vo et al., 2019). In this study, we first analyzed the differentially expressed circRNA in cervical cancer by RNA sequencing. We found that hsa_circRNA_0001400 was highly expressed in cervical cancer tissues and cervical cancer cells. It has been reported that hsa_circRNA_0001400 expression can be induced by viral infection. Therefore, we used hsa_circRNA_0001400_siRNA to interrupt the expression of hsa_circRNA. The cell apoptosis was tested by using flow cytometry. Hsa_circRNA_0001400_siRNA can significantly promote the apoptosis of SiHa and HeLa cells. In SiHa cells, when treated with hsa_circRNA_0001400_siRNA, the apoptosis rate increased from 2.01 to 8.71%, while, in HeLa cells, the apoptosis rate increased from 4.48 to 23.26%. We also analyzed the percentage of cells in each cell cycle. We found that among SiHa cells treated with hsa_circRNA_0001400_siRNA, the cell cycle of G2-phase cells decreased from 5.4 to 4.71%, while in HeLa cells, that of G2-phase cells decreased from 4.9 to 4.6%. In other words, Hsa_circRNA_0001400_siRNA arrested the division of SiHa and HeLa in the G2 phase. Moreover, we identified that hsa_circRNA_0001400_siRNA inhibited the expression of Akt in SiHa and HeLa cells. However, hsa_circRNA_0001400_siRNA did not affect the expression of *PI3K*. These results suggested that the target gene of miRNA adsorbed by hsa_circRNA_0001400 was not *PI3K*.

It is generally believed that one of the important functions of circRNA is to negatively regulate the corresponding miRNA through its binding site, thus affecting its downstream target molecules—namely, to provoke a spongy effect (Weng et al., 2017; Li et al., 2018; Xia et al., 2020b). Among them, the most representative is human circRNA cerebellar degeneration–associated protein 1 transcript (cdr1as)/cirs-7. As an miR-7 sponge, it contains more than 70 miR-7 binding sites, which bind to and regulate the function of miR-7 (Yao et al., 2019; Yin et al., 2019). This study is the first to prove that circRNA has the function of miRNA sponge. However, until 2017, only a few kinds of circRNAs had been proven to be spongy (Yu T. et al., 2019). To verify our hypothesis, we used bioinformatics tools to predict that hsa_circrRNA_0001400 and miR-326 have a sponge effect and we have confirmed the binding sites of hsa_circRNA_0001400 and mir-326 were located at 241 to 247 of the 5′ end of the circular RNA, while the binding target of miR-326 with AKT2 was located at positions 2,224 to 2,234 of AKT2 3′UTR region by luciferase reporter gene analysis. We further verified hsa_circRNA_0001400–miR-326–Akt by means of reversibility experimentation. When we first inhibited hsa_circRNA_0001400 by hsa_circRNA_0001400_siRNA, miR-326 was transformed from the hsa_circRNA_0001400–miR-326 sponge. Specifically, the target gene *Akt* of miR-326 was inhibited, the apoptosis rate of HeLa tumor cells increased from 3.83 to 15.59%, and the number of G1-phase cells decreased from 5.41 to 4.44%. When we used miR-326 inhibitor in combination with hsa_circRNA_0001400_siRNA, the antitumor effect of free miR-326 released from sponge was inhibited, the apoptosis rate of tumor cells decreased from 15.59 to 4.9%, and the number of G2-phase cells increased from 4.44%% to 5.16%. We also detected the protein expression of Akt in SiHa cells and found that, after hsa_circRNA_0001400 was silenced by hsa_circRNA_0001400_siRNA, the expression of Akt decreased, yet it increased when miR-326 inhibitor was added. MiR-326 inhibition can ameliorate the hsa_circRNA_0001400 reduction induced by hsa_circRNA_0001400_siRNA. However after hsa_circRNA_0001400 was silenced by hsa_circRNA_0001400_siRNA in HeLa cells, the expression of Akt increased, yet it decreased when miR-326 inhibitor was added. We did not explain this phenomenon. We also verified the inhibitory effect of hsa_circRNA_0001400_siRNA on the growth of cervical xenograft tumors. In conclusion, we found hsa_circRNA_0001400–miR-326–Akt to be a novel circular RNA sponge, which is involved in tumor metastasis. Hsa_circRNA_0001400_siRNA may be a new treatment for cervical cancer.

At present, most of our research on the role of circRNAs in tumorigenesis and development has been focused on the adsorption function of miRNA sponge by circRNA (Zang et al., 2020). In fact, the mechanism of circRNA in tumor progression is not only one or two aspects. CircRNAs can bind to both mRNA and proteins. Can they act as an intermediary bridge to bring mRNA or protein close to each other and promote the interaction between them, or do they have biological functions as a whole? What are the main components of this complex? The expression level of circRNAs is not high and how circRNAs can effectively compete to bind miRNAs or proteins is a problem that needs to be considered. Although a lot of circRNAs related to tumor metastasis have been identified, most existing studies are limited to including small sample groups or tumor phenotypes and long-term follow-up investigation of clinical information is urgently needed. Therefore, the in-depth and accurate study of circRNA will provide new ideas for researchers to comprehensively explain the molecular mechanism of tumor metastasis and then put forward related methods of tumor-metastasis prevention and treatment. To summarize, circRNAs have unique characteristics, which have significant research value for studying the pathogenesis of diseases, regardless of whether they can be used as diagnostic biomarkers or as therapeutic targets. We need to deepen our understanding of circRNAs and their important functions and mechanisms need to be discovered.

## Conclusion

This study provides evidence that the hsa_circRNA_0001400–miR-326–Akt network promotes cervical cancer progression. Notably, our findings demonstrate the novel antitumor effects of hsa_circRNA_0001400_siRNA in cervical cancer. Since hsa_circRNA_0001400_siRNA play key roles in cervical cancer development and that hsa_circRNA_0001400 might be used as novel diagnostic and prognostic biomarkers as well as for targeted therapies. Furthermore, many studies which establishing circRNAs significance as biomarkers of early disease and clinical outcome indicators are on the way.

## Data Availability

Publicly available datasets were analyzed in this study. This data can be found here: https://www.jianguoyun.com/p/DV5rq5UQi4zqCRjB6o0E Account: xiachenglai@126.com password: Xiacl998.

## Supplementary Material

Supplementary Figure S1 GO analysis was used to analyze **(A)** the biological process of differential expression of circRNA, **(B)** the cell composition of differentially expressed circRNA, and **(C)** the molecular function of differentially expressed circRNA.

## Abbreviations

circRNAs, circular RNAs; HPV, human papillomavirus; miRNA, microRNA; mRNA, messenger RNA; siRNA, small interfering RNA; KEGG, Kyoto Encyclopedia of Genes and Genomes.
