# Supplementary figures and images for: Downregulation of hsa_circRNA_0001400 Helps to Promote Cell Apoptosis Through Disruption of the circRNA_0001400–miR-326 Sponge in Cervical Cancer Cells
Source: Front Genet. 2021 Dec 17;12:779195. doi: 10.3389/fgene.2021.779195 (PMC8718754; doi:10.3389/fgene.2021.779195)

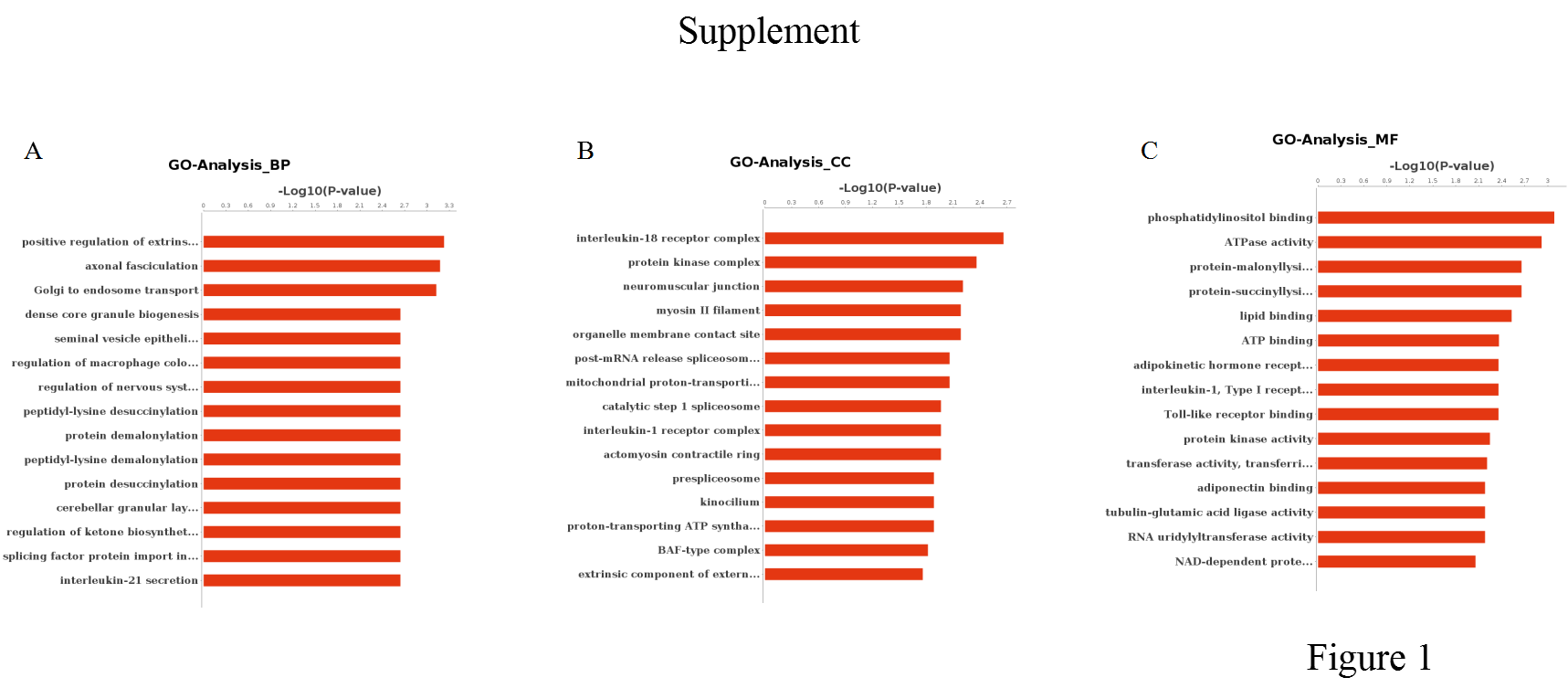

Supplement: Supplementary file 1 [file Image1.TIF]
